# Overweight/obesity and associated cardiovascular risk factors in sub-Saharan African children and adolescents: a scoping review

**DOI:** 10.1186/s13633-020-0076-7

**Published:** 2020-03-24

**Authors:** Simeon-Pierre Choukem, Joel Noutakdie Tochie, Aurelie T. Sibetcheu, Jobert Richie Nansseu, Julian P. Hamilton-Shield

**Affiliations:** 1 Department of Clinical Sciences, Faculty of Medicine and Pharmaceutical Sciences, University of Dschang, Dschang, Cameroon; 2 Health and Human Development (2HD) Research Network, Douala, Cameroon; 3 Diabetes and Endocrine Unit, Department of Internal Medicine, Douala General Hospital, Douala, Cameroon; 4 Department of Anesthesiology and Critical Care Medicine, Faculty of Medicine and Biomedical Sciences, University of Yaoundé I, Yaoundé, Cameroon; 5 Department of Pediatrics, Faculty of Medicine and Biomedical Sciences, University of Yaoundé I, Yaoundé, Cameroon; 6 Disease, Epidemics and Pandemics Control, Ministry of Public Health, Yaoundé, Cameroon; 7 Bristol Biomedical Research Centre (Nutrition Theme), University of Bristol and University Hospitals Bristol NHS Foundation Trust, Bristol, UK

**Keywords:** Obesity, Overweight, Children, Adolescent, Cardiovascular risk factor, Sub-Saharan Africa

## Abstract

**Introduction:**

Recently, childhood and adolescence overweight/obesity has increased disproportionately in developing countries, with estimates predicting a parallel increase in future cardiovascular disease (CVD) burden identifiable in childhood and adolescence. Identifying cardiovascular risk factors (CVRF) associated with childhood and adolescence overweight/obesity is pivotal in tailoring preventive interventions for CVD. Whilst this has been examined extensively in high-income countries, there is scant consistent or representative data from sub-Saharan Africa (SSA).

**Objective:**

This scoping review synthesises contemporary studies on CVRF associated with overweight and obesity in SSA children and adolescents to provide evidence on the current burden of overweight/obesity and CVD in this population.

**Methods:**

We searched MEDLINE and Google Scholar up to July 31, 2019 for observational and experimental studies and systematic reviews addressing childhood and adolescence overweight/obesity and CVRF in SSA without language restriction. Four investigators working in four pairs, independently selected and extracted the relevant data. The methodological quality of all included studies was assessed.

**Results:**

We included 88 studies with a total of 86,637children and adolescents from 20 SSA countries. The risk of bias was low in 62 (70.5%), moderate 18 (20.5%), and high in eight (9%) studies. Overweight/obesity in SSA children and adolescents is rising at an alarming rate. Its main associations include physical inactivity, unhealthy diets, high socio-economic status, gender and high maternal body mass index. Identified CVRF in overweight/obese SSA children and adolescents are mainly metabolic syndrome, hypertension, dyslipidaemia, diabetes and glucose intolerance. There is a dearth of guidelines or consensus on the management of either childhood overweight/obesity or CVRF in overweight/obese SSA children and adolescents.

**Conclusion:**

The current findings suggest an urgent need to review current health policies in SSA countries. Health education and transforming the current obesogenic environment of the SSA child and adolescent into one which promotes physical activity and healthy dietary habits is required.

## Background

Sub-Saharan African (SSA) is one of the poorest regions in the world, with a double public health burden of communicable diseases (malaria, HIV/AIDS, and tuberculosis) and a rising incidence of non-communicable diseases (NCD), especially cardiovascular disease (CVD) [1]. Overweight/obesity is an emerging problem, stemming from increasing urbanisation and westernised lifestyles [2], which in turn has led to the emergence of a nutrition transition characterised by a shift to a higher calorie diet [3]. In this resource-poor setting, the trend in overweight/obesity may likely increase as excess weight is often considered to reflect health, prestige, and prosperity whilst the lean are perceived to be unhealthy or financially poor [4–6].

Likewise, childhood and adolescence overweight/obesity is one of the major global public health problems of the twenty-first century [7, 8]. Globally, the prevalence of overweight and obesity rose by 47.1% in children over the last three decades [7]. Almost 6.7% or 43 million (35 million in developing countries) under-five children were either overweight or obese in 2010, with 92 million at risk of overweight [9]. The global prevalence is expected to reach 9.1% or 60 million in 2020 [9]. Furthermore, the prevalence of overweight and obesity in children and adolescents in developing countries increased from 8.1 to 12.9% in boys and 8.4 to 13.4% in girls over thirty years [7], demonstrating the time trend in this developing epidemic.

The ill-health of childhood and adolescence overweight/obesity is particularly worrisome due to the potential for long-term sequelae in adulthood [10]. Un-addressed, contemporary evidence suggests that overweight/obesity in childhood and adolescence is predictive of an increased risk of adult obesity, cardiovascular, metabolic, psychological complications, some malignancies, and premature death in adulthood [11–14]. Childhood and adolescence associates with hypertension, atherogenic dyslipidaemia, metabolic syndrome, early and accelerated atherosclerosis, type 2 diabetes mellitus and obstructive sleep apnoea [15–19]. Although investigating CVD risk factors among children and adolescents is crucial because childhood or adolescence is a critical temporal window for the development of obesity in adulthood [20], these risk factors have been less well examined in overweight/obese SSA children and adolescents. This review critically synthesises the current burden of childhood and adolescence overweight/obesity and associated cardiovascular risk factors, as well as contemporary diagnostic and therapeutic options in SSA. This should inform policy-makers on the various public health interventions necessary to mitigate cardiovascular risk in this vulnerable overweight/obese population at these earlier stages.

## Methods

**Fig. 1:**
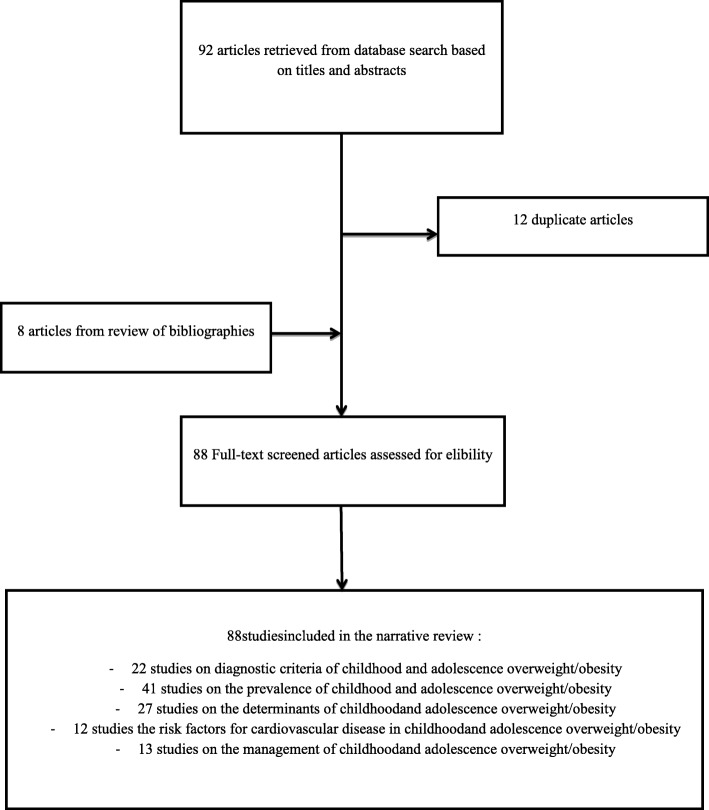
Flow diagram of study selection

We searched two main electronic databases: MEDLINE (via Pubmed) and Google Scholar from inception to July 31, 2019 for observational, interventional studies and systematic reviews addressing childhood and adolescence overweight/obesity and cardiovascular risk factors in SSA without language restriction. A comprehensive search strategy was conducted using the key words: “overweight”, “obesity”, “children”, “adolescent”, “prevalence”, “cardiovascular risk factor”, “diagnosis”, “management”, cross-referenced with sub-Saharan Africa or the names of sub-Saharan Africa countries to obtain the maximum possible number of studies (Table 1). The reference lists of retrieved articles were scanned in order to identify any additional relevant study. Eligible articles and documents were scrutinised based on adequate sample size and robust study design to extract data on the prevalence of childhood and adolescence overweight/obesity, associated cardiovascular risk factors, diagnostic methods and treatment strategies in sub-Saharan Africa. One pair of investigators (SPC and JNT) independently screened records by abstract and title. Subsequently, two pairs of investigators (SPC, JNT, ATS and JRN) independently screened possibly relevant full texts for articles directly reporting the definition, prevalence, management of overweight/obesity in SSA as well as CVRF associated with overweight and obesity in SSA children and adolescents. We only included peer-reviewed cross-sectional, case-control, cohort studies, randomized controlled trials and systematic reviews recruiting at least 30 SSA children and adolescents residing in SSA. We excluded letters to the editor, modeling studies, qualitative studies, and conference proceedings. Studies reporting overweight/obesity linked to corticosteroids, eating disorders, family hypercholesterolemia, antipsychosis medication were excluded. Missing data was sought by contacting the corresponding author of the research article via emails. When the corresponding author could not be contacted, the article was excluded. Discrepancies between two pairs of investigators (SPC, JNT, ATS and JRN) were solved through discussion and consensus. A standardized and pre-tested data extraction form was used by two pairs of investigators (SPC, JNT, ATS and JRN) to independently extract bibliometric information (the name of first author), study characteristics (country and sample size), participants’ age range, diagnostic criteria for overweight/obesity, prevalence of overweight, obesity and combined overweight/obesity. Data from each country was reported separately for multinational studies. Study quality for observational, randomized controlled trials and systematic reviews was assessed using the methods described by Hoy et al. [21], the SPIRIT 2013 Statement tool [22] and the AMSTAR 2 tool [23] respectively. Finally, using data retrieved from a myriad of epidemiological studies, interventional studies and systematic reviews, the ensuing findings present a narrative synthesis of the most up-to-date and key literature regarding childhood and adolescence overweight/obesity and cardiovascular risk factors in SSA (Fig. 1).

**Table 1:** Search strategy for MEDLINE and adaptability to Google scholar data base

Region/Country	sub Saharan Africa OR sub Saharan African OR subSaharan Africa OR Angola OR Benin OR Botswana OR Burkina Faso OR Burundi OR Cameroon OR Cape Verde OR Central African Republic OR Chad OR Comoros OR Congo OR Democratic Republic of Congo OR Djibouti OR Equatorial Guinea OR Eritrea OR Ethiopia OR Gabon OR Gambia OR Ghana OR Guinea OR Guinea-Bissau OR Ivory Coast OR Kenya OR Lesotho OR Liberia OR Madagascar OR Malawi OR Mali OR Mauritania OR Mauritius OR Mozambique OR Namibia OR Niger OR Nigeria ORPrincipe OR Reunion OR Rwanda OR Sao Tome OR Senegal OR Seychelles OR Sierra Leone OR Somalia OR South Africa OR Sudan OR Swaziland OR Tanzania OR Togo OR Uganda ORWestern Sahara OR Zambia OR Zimbabwe OR Central Africa OR Central African OR West Africa OR West African OR Western Africa OR Western African OR East Africa OR East African OR Eastern Africa OR Eastern African OR South African OR Southern Africa OR Southern African.
Disease/Risk factor	Cardiovascular risk factor OR hypertension OR high blood pressure OR elevated blood pressure OR salt intake OR diabetes OR artherosclerosis OR glucose intolerance OR dyslipidemia OR cholesterol OR triglyceride OR smoking OR tobacco OR alcohol consumption OR physical inactivity OR lack of exercise OR diet OR nutrition OR urbanization OR socio-economic status OR lack of sleep OR sleep apnoea.
Participants	Children OR child OR childhood OR infants OR toddlers OR adolescents OR adolescence OR obesity OR obese OR overweightOR nutritional status OR fat OR fatness OR adiposity OR fatty OR body size

## Results and discussion

**Selection**: We included 88 studies with a total of 86,637 children and adolescents from 20 SSA countries. The risk of bias was low in 62 (70.5%), moderate in 18 (20.5%), and high in eight (9%) studies.

### Clinical assessment and diagnostic criteria

The assessment of childhood and adolescence overweight/obesity has been a subject of debate in SSA due to the absence of universal consensus [24]. Although fat mass and body mass index (BMI) among children and adolescents are often positively correlated [25, 26], the assessment of childhood and adolescence overweight/obesity using BMI may be flawed by several draw backs: BMI does not discriminate between lean and fat mass; BMI varies sharply with respect to growth, gender, and ethnicity [27, 28]; BMI alone is argued to have high specificity but low sensitivity to detect excess adiposity [29]. As a result, international cut-offs [30–33] using percentiles and standard deviations from a median reference point are preferred (Table 2) to traditional BMI cut-off values of ≥30 kg/m2 and ≥ 25 kg/m2 for obesity and overweight, respectively. The dual use of the International Obesity Task Force (IOTF) and WHO cut-offs is recommended for the assessment of the prevalence of childhood and adolescence overweight [34].

**Table 2:** International cut-off values for childhood overweight and obesity

International cut-offs	Age group	Cut-off
WHO growth standard [27, 28]	< 5 year	Overweight: + 2SD ≤ BMI < +3SD Obesity: BMI ≥ + 3 SD
	5–19 years old	Overweight: +1SD < BMI < + 2SD Obesity: BMI > +2 SD
Centre for Diseases Control [29]	2–19 years old	Overweight = 85th – 94th BMI percentiles Obesity >95th BMI percentiles
International Obesity Task Force [30]	2–18 years old	Overweight percentile curve passing though BMI = 25 kg/m2 at age 18; Obesity percentile curve passing through BMI = 30 kg/m2 at age 18

SD: Standard deviation; BMI: Body Mass Index for age and sex

In a recent systematic review of 283 articles investigating overweight/obesity transition in SSA school-aged children, international cut-offs were reported in 82 articles (29%) as the scale used to stratify body weights into underweight, normal-weight, overweight and obesity [24]. Meanwhile, 48.8% of included studies cited the mean BMI, BMI-z-score, weight z-scores, body fat percentage, waist circumference, skin fold measures, and/or weight and height measures as the diagnostic tools for childhood and adolescence overweight/obesity [24]. The high heterogeneity of the types of measurements altered the comparability of studies, and regional time trends analyses. Thus, there is a need for a universal consensus towards scales such as WHO cut-points which have the merit of being more sensitive to identify overweight/obese male and female children irrespective of the age groups [35].

### Prevalence and trends of overweight and obesity in SSA

#### Prevalence in preschool children (under-five years)

Evidence from a recent meta-analysis of demographic and health surveys from 26 SSA countries suggest that 10.7 million or 6.8% of under-five children were overweight or obese between 2010 and 2014 [36]. Countries with alarming prevalence levels of childhood and adolescence overweight/obesity included Sierra Leone (16.9%), Cameroon (15.9%) and Malawi (14.5%), while much lower prevalence was observed in Ethiopia (3.0%), Togo (2.6%) and Senegal (2.0%) [36]. On the other hand, there is a parallel increase in the burden of stunting in Africa, a proof of the on-going dual burden of over- and under-nutrition in the continent [37].

#### Prevalence in school-aged children and adolescents

There is a high heterogeneity in studies reporting prevalence levels of overweight/obesity in school-aged children in SSA due to the large socio-cultural differences, the rapidity of the epidemiologic transition and methodological differences between studies [24]. As of the year 2013, results from a meta-analysis based on SSA countries showed that prevalence of overweight and obesity in SSA school-aged children (5–17 years) was 10.6 and 2.5%, respectively [24]. More representative national data on the prevalence of childhood overweight/obesity in SSA [38–73] is illustrated in Table 3. Although these prevalence levels are high, they are lower than trends in childhood and adolescence obesity in high-income settings. For instance, the prevalence of overweight and obesity in the USA is 33 and 18%, respectively [74]. Similarly, over the past 25 years, the prevalence of overweight/obesity has doubled (14 to 29%), while the obesity rate has tripled (3 to 9%) in both Canadian children and adolescents [35, 75].

**Table 3:** Prevalence of overweight/obesity in school-aged children for different sub-Saharan African Countries

Survey year	Investigators	Sub-Saharan African Country	Sample Size	Participants’ Ages (years)	Diagnostic criteria	Prevalence rates (%)		
						Overweight	Obesity	Combined overweight/obesity
2009	Manyanga T et al [39]	Benin	2681	13–17	WHO	11.2	0.6	11.8
2008–2009	Dabone C et al [40]	Burkina Faso	649	7–14	WHO	N/A	N/A	2.3
2010	Koueta F et al [41]	Burkina Faso	435	13–25	IOTF	N/A	N/A	8.6
2013	Choukem SP et al [42]	Cameroon	1343	3–13	WHO	9.6	2.9	12.5
2007	Manyanga T et al [39]	Djibouti	1711	13–17	WHO	18.8	5.2	24
2012	Teshome T et al [43]	Ethiopia	559	10–19	WHO and TSFT	11–12.9	2.7–3.8	N/A
2013	Alemu E et al [44]	Ethiopia	800	15–19	CDC	8.6	0.8	9.4
2014	Askal T et al [45]	Ethiopia	845	9–14	CDC	8	1.8	9.8
2014	Gebremichael B et al [46]	Ethiopia	463	10–18	CDC	9.9	2.8	12.7
2014	Shegaze M et al [47]	Ethiopia	456	13–19	WHO	9.7	4.2	13.9
2016	Desalew A et al [48]	Ethiopia	448	11–15	CDC	14.7	5.8	20.5
2006	Manyanga T et al [39]	Ghana	6156	13–17	WHO	8.7	1.0	9.7
2008	Kyallo F et al [49]	Ghana	344	9–14	WHO	N/A	N/A	19
2010	Morge V et al [50]	Ghana	218	5–14	WHO	N/A	N/A	17.4
2010	Kumah DB et al [51]	Ghana	500	10–20	IOTF	12.2	0.8	13
2012	Mohammed H et al [52]	Ghana	270	5–15	WHO	15.8	10.9	26.7
2010	Kramoh KE et al [53]	Ivory Coast	2038	6–18	BMI	4	5	9
2011	Kamau JW et al [54]	Kenya	5325	10–15	BMI	8.7	3.1	11.8
2010	Van den Berg VL [55]	Lesotho	221	16	WHO, CDC and IOTF	10.4–15.4	1.8–4.1	14.5–19
2009	Manyanga T et al [39]	Malawi	2305	13–17	WHO	10	0.8	10.8
2014	Oumar H et al [56]	Mali	984	5–19	WHO and IOTF	2.6–5.12`	0.3–1.8	N/A
2010	Manyanga T et al [39]	Mauritania	2028	13–17	WHO	24.3	3.4	27.7
2006	Caleyachetty R et al. [57]	Mauritius	841	9–10	IOTF	17.4	4.9	22.3
1983–2013	Ejike CECC [58]	Nigeria	21842⃰	3–20	WHO, IOTF, TSFT and BMI	5–12	0–5.8	N/A
2015	Adam VY et al [59]	Nigeria	195	6–12	WHO	7.7	3.1	10.8
2011	Faye J et al [60]	Senegal	2356	11–17	N/A	N/A	9.34	N/A
1999	Stettler N et al [61]	Seychelles	5514	4–17	IOTF	12.6	3.8	16.4
2001–2004	Armstrong MEG et al [62]	South Africa	10,195	6–13	CDC	15.8	3.9	19.7
2010	Toriola AL et al [63]	South Africa	1172	10–16	CDC	10.1	4.9	15
2011	Tathiah N et al [64]	South Africa	963	9–12	CDC	9	3.8	12.8
2013	Pienaar AE [65]	South Africa	547	6–9	CDC	9.4	7.3	16.7
2007	Aisha AMB et al [66]	Sudan	80	5–13	CDC	18.75	18.75	37.5
2011	Nagwa MA et al [67]	Sudan	1138	10–18	WHO	10.8	9.7	20.5
2011	Salman Z et al [68]	Sudan	304	6–12	CDC	14.8	10.5	25.3
2015	El Raghi HA et al [69]	Sudan	290	10–18	BMI	26.2	28.3	54.5
2012	Pangani IN et al [70]	Tanzania	1781	8–13	WHO	15.9	6.7	22.6
2015	Kimario JT [71]	Tanzania	140	10–12	IOTF, TSFT	N/A	N/A	20–24.3
2014	Chebet M et al [72]	Uganda	958	8–12	BMI	32.3	21.7	54
2013	Nsibambi CAN [73]	Uganda	1929	6–9	WHO and CDC	7	4	11
2011	Peltzer K et al [74]	Uganda and Ghana	5613	13–15	BMI	6.19	0.71	6.9

*Total sample size of narrative review of 42 studies conducted in Nigeria. BMI: Body mass index; CDC: Centres for Disease Control and Prevention; IOTF: International Obesity Task Force; N/A: Not available data; TSFT: Triceps skinfold thickness; UN: United Nations; WHO: World Health Organisation

### Factors associated with overweight/obesity and cardiovascular diseases in sub-Saharan African children and adolescents

Several risk factors in obese/overweight SSA children and adolescents have been recognised to contribute to the development of CVD. These include determinants of childhood and adolescence obese/overweight on the one hand and risk factors for the development of CVD among overweight/obese children and adolescents on the other hand.

#### Determinants of childhood and adolescence overweight/obesity in sub-Saharan African children and adolescents

##### Gender and age

The sex distribution of obesity in SSA has a predilection for the female gender. In SSA, on average, 7.6% of boys and 15.4% of girls aged 5–17 years are overweight/obese, while the prevalence of obesity in boys and girls aged 5–17 years is 2.0 and 3.9%, respectively [24]. The reverse is true for the under-fives as supported by findings of a meta-analysis which identified that boys are 1.15-fold more likely to be overweight/obese compared to girls in SSA [36]. Higher trends of overweight/obesity in SSA girls may be explained by differences in gender roles especially those necessitating strenuous physical activity more among boys than girls [76], and social/cultural desirability whereby being overweight/obese is an admired trait for girls [5].

##### Physical inactivity

The rise in the prevalence of childhood and adolescence overweight/obesity has also been linked with an increase in childhood and adolescence sedentary behaviours, mainly due to indoor activities such as computer games, television viewing, and the internet [42, 45, 47, 77, 78]**.** In Ghana, a cross-sectional study among senior high school students aged 15 to 19 years, showed a significant association between physical inactivity and overweight [76]. More recently, a similar trend was observed in a younger cohort (3–13 years) of 1343 Cameroonians demonstrating a positive association between overweight/obese status and regular utilisation of passive means of travel to school or not doing sport at school [41]. Similarly, a recent Ethiopian cross-sectional study reported that physical activity conveyed protection (adjusted odds ratio [AOR]: 0.21, 95% confident interval [95% CI]: 0.08–0.57) against child and adolescent overweight/obesity while children who spent ≥3 h per day sitting, increased their odds for overweight/obesity by 3.5 [46]. A composite of factors contribute to physical inactivity of overweight/obese children in low-income settings like SSA: urbanisation of cities with resultant lack of open playgrounds in schools and communities, the increase in criminal acts which render neighbourhoods unsafe for outdoor activities, and the persistent emphasis on academic excellence at the expense of physical activity of children [79].

##### Unhealthy diet

Regular consumption of an unhealthy diet is a major precursor for obesity, metabolic syndrome, type 2 diabetes and coronary artery disease. Developing countries, including those in SSA, are not immune to this risk considering the shift from consumption of traditional low-energy density to high-calorie westernized foods [45, 80]. This type of diet is rich in refined carbohydrates, saturated fat and sweetened carbonated beverages, with low levels of polyunsaturated fatty acids and fibres [39, 41]. More interestingly, the consumption of sweet foods [40, 42, 45–47], skipping breakfast [45], eating more than three regular meals per day [46] and eating two snacks per day [45], have been identified as independent determinants of childhood and adolescence overweight/obesity in SSA as in other parts of the World. Children and adolescents are particularly exposed because of the sale of ‘fast foods’ in school cafeteria [81]. Aggressive advertisement by multinational companies and lack of public awareness of the health effects these high-energy foods contribute to obesity risk in children [82]. Furthermore, prolonged television/computer viewing hours decreases the time allocated to physical activity whilst favouring the passive consumption of junk foods and sweetened beverages. In addition, children and adolescents in particular are exposed to advertisements of these unhealthy foods in the media [83]**.** In the Birth-to-twenty cohort conducted in South Africa, eating patterns of participants aged 13 and 17 years were evaluated and revealed that consumption of snacks while watching screens was common and significantly increased with age [83]**.** In South African school premises, 85% of adolescents purchase food and 62% are unhealthy like candies, crisps, cold drink, fried chips, and white bread [83]**.**

##### Socio-economic status (SES)

In developed countries, high SES is inversely related to child and adolescent adiposity [79]**.** However, the reverse is true in low- and middle-income countries [40, 42, 64, 84]. We found two systematic reviews pointing to a positive association between overweight/obesity and SSA children of higher SES [24, 85], probably due to increased sedentary behaviours and increased accessibility to packaged foods high in sugars and saturated fats, more affordable to families with of higher SES or living in urban settings. Recently, a Cameroonian study investigating the association between childhood overweight/obesity and SES found that children from high SES families were two-fold more likely to be overweight/obesity than children from families with low SES [41]. Interestingly, the risk of overweight/obesity for children of high SES persisted after adjusting for potential confounders like age, gender, early life factors (birth weight and type of feeding till six months of age), parental factors (maternal age, BMI, alcohol consumption, maternal and paternal education level) and current child factors (number of meals per day, consumption of fruits and sweet drinks, physical activity at school and leisure time, travel means to school, television and electronic use habits, sleeping habits and pocket money) [41].

##### Urban versus rural residence

Several studies, including systematic reviews, have clearly shown that childhood overweight/obesity is more prevalent in urban than in rural settings of SSA [24]. In a study involving 1799 Nigerian adolescents aged between 10 and 19 years, the comparison of BMI between adolescents of rural and urban areas revealed higher BMI amongst the latter [86]**.** Similar data were reported in a nationwide study carried out in Cameroon, where urban children aged 6–59 months had a higher prevalence of overweight/obesity than their counterparts in rural settings (8.7% vs. 7.6%) [87]. As stated previously, the reasons may stem from urbanisation of SSA cities and an economic transition which in turn has led to an increased sedentary behaviour and an easy access to packaged foods high in saturated fats and sugars, two major risks factors for obesity [24].

##### Pre-conception, maternal, and intrauterine factors

There is limited data citing the impact of maternal pre-gestational and gestational BMI on body composition of off-spring in SSA. The few available studies highlight that overweight/obese mothers have a 1.07 to 2 fold increased risk of having an overweight/obese child [36, 60, 87, 88].

Maternal level of education has also been described as predictive of offspring overweight and obesity. Children whose mothers have primary education or no formal education have been reported in a meta-analysis to respectively have a 1.23 and 1.10 times increased odds of being overweight/obese [36], probably explained by the fact that less educated mothers may have poor knowledge on healthy diets or may perceive childhood overweight/obesity as healthy and desirable. In contrast, findings from a Kenyan study not included in this meta-analysis identified that primary, secondary and higher maternal levels of education were associated with higher odds of childhood overweight/obesity [88]. This could be explained by the fact that an educated mother is more likely to be employed and have a higher household income, which in turns may lead to increased affordability for high energy-dense foods. Likewise, the prevalence of childhood overweight/obesity in SSA has been reported to increase with decreasing maternal age [36].

##### Birth weight

One systematic review [36] and two cross-sectional studies [87, 88] have shown higher odds of overweight/obesity for SSA children born with the traditional cut-off value ≥4000 g for high birth weight (HBW) (≥ 4000 g) in SSA. However, using the standard recommended WHO definition for HBW (90th centile of birth weights), a more recent study conducted on almost 5000 neonates in a SSA setting, found 3850 g to be the actual cut-off of HBW [89]. Hence, the aforementioned studies [36, 87, 88] may have some methodological flaws in that they underestimated the true association between childhood overweight/obesity and HBW due to the fact that the exact 90th centile of birth weights was not considered.

##### Miscellaneous determinants

Low birth order has also been associated with a lower risk of obesity and overweight in SSA [36]. This relationship may be secondary to the higher susceptibility of first order births to low birth weight, which in turn can result in lower weight during childhood [90]. Also, the association across religions has been reported in a nationwide Cameroonian study [87]. The Muslim religion was associated with a lower risk of childhood overweight and obesity compared to Christianity. According to the authors, the dietary habits can be influenced by the compliance to the rules of religion [87]. In addition, learning in a private school has been shown to increase the odds of overweight/obesity in Ethiopian [43, 46] and Burkinabe children and adolescents [39]. However, this risk is confounded by SES, given that students enrolled in private schools are usually of high SES which exposes them to more high caloric foods and passive means of transport to school compared to students attending government schools.

#### Risk factors for the development of cardiovascular disease among overweight/obese children and adolescents

##### Metabolic syndrome

Paediatric metabolic syndrome is defined as the constellation of at least three of the following criteria: (i) fasting glucose ≥110 mg/dl; (ii) high density lipoprotein cholesterol ≤50 mg/dl (except in boys aged 15 to 19 years in whom the cut point is 45 mg/dl); (iii) fasting triglyceride ≥100 mg/dl; (iv) systolic blood pressure > 90th percentile for gender, age and height; (v) waist circumference > 75th percentile for age and gender [91]. Other more universally accepted definitions include that of the International Diabetes Foundation which defines paediatric metabolic syndrome as an association of at least two metabolic abnormalities (central obesity ≥9Oth percentile, high blood pressure ≥ 130/85 mmHg, hypertriglyceridemia ≥150 mg/dl or 1.7 mmol/l, low high density lipoprotein cholesterol below 40 mg/dl or 1.03 mmol/l, impaired glucose tolerance with glycaemia ≥100 mg/dl or 5.6 mmol/l) [92]. Obesity is the major trigger of this syndrome since it is well known that being overweight during childhood is associated with glucose intolerance, high levels of serum lipids and elevated blood pressure in young adulthood [37]**.** Insulin resistance, secondary to central adiposity has multiple metabolic consequences other than dysglycaemia has multiple metabolic consequences other than dysglycaemia including enhanced cholesterol synthesis, increased high-density lipoprotein (HDL) degradation, increased sympathetic activity and proliferation of vascular smooth muscle cells [93]**.**

There is a parallel increase between the prevalence of metabolic syndrome and childhood overweight/obesity in SSA [94]. In a case-control study conducted on 10–16 years old South African children and adolescents, the prevalence of metabolic syndrome in obese students was 13.2–30.2% depending on the definition used. Low HDL was the most common (48.3%) component of paediatric metabolic syndrome whilst impaired fasting blood glucose the least frequent (5.6%) [95]. In a recent cross-sectional study conducted in Cameroon to compare the cardio-metabolic profile of obese children versus matched lean control subjects, the prevalence of metabolic syndrome was 19% [96]. A higher prevalence of elevated blood pressure, dyslipidaemia and type 2 diabetes was found in obese children compared to lean counterparts [96].

##### Diabetes, insulin resistance, and glucose intolerance

Data on glucose intolerance and type 2 diabetes in overweight/obese SSA children arescarce. In the aforementioned cohort of 38 obese Cameroonian children aged 3 to 17 years, only one child had type 2 diabetes [96]. However, 60% of these obese children had acanthosis nigricans, a marker of insulin resistance [96]. If not addressed, the prevalence of type 2 diabetes in this population will certainly increase. In a retrospective study to determine the prevalence of type 2 diabetes among 985 Sudanese children and adolescents, type 2 diabetes was found in 38 (4%) children and adolescents who were all obese [97].

##### Hypertension

There is also little data on the prevalence of hypertension in overweight/obese SSA children and adolescents. Hypertension was strongly associated with obesity in a study involving South African adolescent aged 13–17 years [98]. The prevalence of hypertension (systolic and diastolic blood pressure ≥ 95th percentile for age, sex and height) and prehypertension (systolic or diastolic blood pressure ≥ 90thto < 95thpercentile for age sex and height) in obese adolescents was 32.6% in females and 32.8% in males [98]. Meanwhile, in Cameroon, the prevalence of hypertension in obese children was 25% [96] and 27.7% of obese Nigerian adolescents were hypertensive [99]. Furthermore, the prevalence of hypertension in overweight and obese Sudanese children was 17.8 and 31.2% respectively [67]. Obese Sudanese children were 15-fold more likely to have systolic hypertension than normal-weight counterparts [67].

##### Atherogenic dyslipidaemia, atherosclerosis and smoking

We identified one study in Cameroon, which demonstrated that the prevalence of dyslipidaemia (high levels of total cholesterol, low-density lipoprotein (LDL) cholesterol and triglycerides) in obese children was 16% [96]. There was a statistical significant difference in the medians of total cholesterol (164.5 vs. 115 mg/dl) and LDL cholesterol (102.5 vs. 72 mg/dl) between obese and lean Cameroonian children [96].

A study on Ghanaian and Ugandan adolescents aged 13–15 years demonstrated a positive association between overweight/obesity and smoking [73]. Here, smoking increased the odds of overweight/obesity by 1.75 and 1.52 in regular male and female adolescent smokers, respectively [73]. However, because the study used a cross-sectional design, it was impossible to infer causality or untangle bi-directional relationships.

**Fig. 2:**
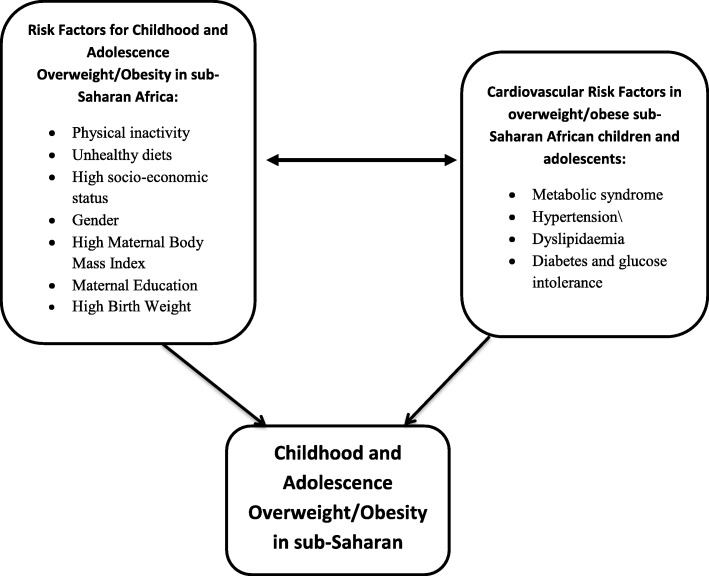
The interaction between childhood and adolescence obesity or overweight and cardiovascular risk factors in sub-Saharan Africa

Overall, Fig. 2 illustrates the interaction between childhood and adolescence obesity or overweight and cardiovascular risk factors in sub-Saharan Africa 11.

### Management of childhood and adolescence overweight/obesity

Currently, there have been no clinical trials performed in SSA to ascertain which treatments may be best suited for preventing or reducing childhood and adolescence overweight/obesity and its associated cardiovascular risk factors. However, it is also true that interventions from mainly high-income settings comparing that combined behavioural interventions (physical activity, dietary therapy) to standard or no care can yield a statistically significant but probably clinically insignificant reduction in body mass indices in children and adolescents [100–102].

Strategies for overweight/obesity prevention and treatment in children and adolescents should be implemented holistically in natural settings in order to impact on diet and physical activities in preschool, schools, after-school care services and at homes. The management of childhood and adolescence overweight/obesity requires a multi-sectoral public health approach that focuses on transforming the current obesogenic environment of the child into one which promotes physical activity, healthy diet and the early recognition of excessive weight gain relative to linear growth [103, 104].

#### Physical activity

Although there is no high-quality study investigating the impact of physical activity on overweight/obese children and adolescents with cardiovascular risk factors in SSA, guidelines from similar resource-limited settings recommend that overweight/obese children and adolescents should engage in at least 45–60 min of moderate intensity physical activity (either sports or bicycling) most days of the week [105]. Meanwhile, WHO guidelines stipulate that obese children and adolescents aged between 5 and 17 years old should carry out a minimum of 60 min of moderate-to-vigorous intensity physical activity at least three times per week [106]. This measure yields fundamental health benefits such as increased cardiorespiratory fitness, muscular strength, reduced body fat, enhanced bone health, favourable metabolic biomarkers, and reduced symptoms of anxiety and depression [106]. Physical activities should preferably be aerobic and sessions of more than 60 min duration have been shown to provide additional health benefits [106]. The type of physical activity ought to be individualised according to the preference and interest of the child, culture, and local practices. For instance, aerobic dance with popular music may appeal more to girls, whereas boys may enjoy more vigorous outdoor sport and martial arts. The involvement of parents is equally pivotal in determining the success rate of the chosen physical activity [107, 108].

#### Healthy diets

Public health interventions to tackle childhood and adolescence overweight/obesity in SSA through healthy eating are few. Currently, only South Africa has school-based interventions to promote healthy eating habits in overweight/obese primary-school children, with a main focus on children from low socio-economic status [109, 110]. These public health programs have modules on physical activity, complications of cigarette smoking and chronic diseases (especially type 2 diabetes). Here, the adherence of children is enhanced through the concomitant involvement of teachers and parents in all modules [109, 110]. Furthermore, it is important to limit the availability of high energy density fast foods (chocolates, candies, ice creams, patties, potatoes or plantain chips) and carbonated beverages in school refectories while simultaneously providing healthier options for children [79].

Strategies to curb the burden of childhood and adolescence overweight/obesity through eating behaviours could be extended to homes because children and adolescents partaking in regular family meals are more likely to eat vegetable- and fruit-based diets and less likely to consume unhealthy foods [111]. Unlike non-vegetarians, vegetarian children and adolescents are usually of normal BMI and this BMI advantage increases during adolescence [112]. This is probably because vegetable-based diets are of low-caloric density, but high in complex carbohydrate, fibre and water, which consequently increase satiety and resting energy expenditure. Also, healthy cooking methods entailing boiling, steaming, roasting, and baking should be adopted by parents/guardians of children and adolescents. Finally, the ideal way for parents to encourage healthy eating habits in children is by setting an example. Parents should share at least one meal with children, preferably a balanced diet. They should themselves have smaller portions, fruits, and vegetables whilst discouraging overeating in their children [79].

## Conclusion

This review examined the problems associated with childhood and adolescence overweight/obesity and cardiovascular risk factors in SSA. These may be resolved if the following recommendations are put in place. Firstly, we need to establish a more robust clinical method to assess childhood and adolescence overweight/obesity in SSA through the universal adoption of the WHO cut-off points. This possibly should be accompanied by a basic screening of cardiovascular risk factors in children and adolescents found overweight or obese: blood pressure, basic lipid profiles and where age appropriate smoking history. Secondly, there is urgent need to decrease the high prevalence of childhood and adolescence overweight/obesity; accordingly, health promotion activities need to be instituted in SSA through health education of parents/guardians, children and adolescents on the ill-health of childhood and adolescence overweight/obesity. Thirdly, there is the need to revamp the current educational system in SSA countries through the incorporation of a compulsory module on physical activity and healthy diets in schools. Furthermore, the involvement of parents/guardians in these interventions will be important to enhance children’s adherence. In addition, state taxation on energy dense food and sugary drinks and a ban on the advertisement of high energy dense foods like fast foods, sweet foods, and beverages by food industries will decrease the number of SSA children and adolescents purchasing or having access to these products. Finally, SSA ministries of health should work in collaboration with ministries of education to ensure cost-effective implementation of these public health interventions. Overall, these policies will go a long way to build sustainable health and educational systems that could help in reducing the burden associated with this condition in SSA.

## Data Availability

All data generated or analysed during this study are included in this published article.

## Abbreviations

BMI: Body mass index
CDC: Centres for Disease Control and Prevention
CVD: Cardiovascular disease
CVRF: Cardiovascular risk factor
HDL: High-density lipoprotein
IOTF: International Obesity Task Force
LDL: Low-density lipoprotein
NCD: Non-communicable disease
SES: Socio-economic status
SSA: sub-Saharan Africa
UN: United Nations
WHO: World Health Organisation
